# Cytotoxicity of Endocytosis and Efflux Inhibitors in the BeWo Cell Line

**DOI:** 10.9734/JPRI/2017/34606

**Published:** 2017-07-01

**Authors:** Mansi Shah, Luke Bourner, Shariq Ali, Sanaalarab Al-Enazy, Erik Rytting

**Affiliations:** 1 Department of Obstetrics and Gynecology, University of Texas Medical Branch, 301 University Boulevard, Galveston, TX 77555, USA; 2 Department of Pharmacology and Toxicology, University of Texas Medical Branch, 301 University Boulevard, Galveston, TX 77555, USA

**Keywords:** Cell viability, cytotoxicity, BeWo cells, endocytosis, efflux, placenta

## Abstract

**Aims:**

The purpose of this study was to determine the cell viability and cytotoxicity of various endocytosis and efflux inhibitors which can be used to determine transport and uptake mechanisms in the BeWo (b30 clone) human placental trophoblast cell line. Ethanol and dimethylsulfoxide (DMSO) were also studied since they are often used as cosolvents for administration of these inhibitors.

**Methodology:**

The water-soluble tetrazolium-1 (WST-1) assay was used to quantify cell viability and the lactate dehydrogenase (LDH) assay was used to determine cytotoxicity.

**Results:**

By the WST-1 assay, reduced cell viability was observed following 4 hours of exposure to chlorpromazine (10 μg/mL), colchicine (1 mM), filipin (3 μg/mL), gentamicin (2 mM), GF120918 (1 μM), methyl-β-cyclodextrin (5 mM), and verapamil (100 μM). By the LDH assay, however, no cytotoxicity was observed after 4 hours of exposure to the aforementioned compounds. Amiloride (500 μM), ethanol (up to 0.1% v/v), and DMSO (up to 0.1% v/v) did not reduce cell viability nor induce cytotoxicity.

**Conclusion:**

This information is valuable when selecting potential inhibitors of endocytosis and efflux and the selection of time points for mechanistic studies.

## 1. INTRODUCTION

Although pregnant women are often excluded from clinical trials, many medications are still prescribed during pregnancy. The placental transport of these drugs and their effects upon fetal health and development are of concern. Several experimental models have been established to study the transplacental transfer of drugs [1]. The BeWo b30 cell line is a placental trophoblast cell line of human origin that can form confluent monolayers to study the transport and metabolism of drugs across the human placental barrier [2,3].

Various endocytosis and efflux inhibitors can be used to elucidate transport mechanisms [4]. However, the effect of incubation time on the cytotoxicity of these inhibitors in BeWo cells has not been investigated previously to the best of our knowledge. If these inhibitors are toxic to cells, it could result in misinterpretation of the mechanistic data obtained. Therefore, we examined the effect of those inhibitors on BeWo cells, as well as the effects of ethanol and DMSO, which are common cosolvents used to solubilize those and other compounds.

To determine cell viability and cytotoxicity mediated by inhibitors and solvents, we used the water-soluble tetrazolium-1 (WST-1) assay and the lactate dehydrogenase (LDH) assay, both of which are commonly used colorimetric assays for cytotoxicity, but which function by different mechanisms. In the WST-1 assay, soluble formazan dye is formed from the stable tetrazolium salt WST-1 at the cell surface due to the glycolytic production of NAD(P)H in viable cells. Therefore, a higher amount of formazan dye formation, measured by absorbance, indicates higher metabolic activity in the cells [5]. In contrast, the measurement of LDH activity in the cell culture supernatant is an indication of cytotoxicity. LDH released into media causes the reduction of NAD^+^ to NADH/H^+^ which can be used in the diaphorase-catalyzed reduction of a tetrazolium dye to a formazan product, which is measured by absorbance. Higher LDH enzymatic activity in the cell culture supernatant corresponds to an increase in the number of dead or plasma membrane-damaged cells [6]. Therefore, an increase in LDH activity is directly correlated with higher cytotoxicity.

## 2. MATERIALS AND METHODS

### 2.1 Inhibitors

A number of inhibitors were investigated as potential candidates to elucidate mechanisms of endocytosis or efflux in placental trophoblast cells for drugs or drug carriers that could be used during pregnancy. The cytotoxicity of these inhibitors was investigated at concentrations specified in the literature for inhibition of particular mechanisms. To elucidate the absorption mechanism, different endocytosis inhibitors were used. For our experiments, we have used amiloride as an inhibitor of micropinocytosis [7], chlorpromazine as an inhibitor of clathrin-mediated endocytosis [8], colchicine as an inhibitor of micropinocytosis [4], filipin as a lipid raft inhibitor [9], gentamicin as an inhibitor of megalin [10], methyl-β-cyclodextrin as a lipid raft inhibitor [9], as well as GF120918 [11] and verapamil [12] as efflux inhibitors.

Amiloride hydrochloride was obtained from Alfa Aesar (Ward Hill, MA, USA). Chlorpromazine hydrochloride and colchicine were purchased from Chem-Impex International, Inc. (Wood Dale, IL, USA). Filipin complex from Streptomyces filipinensis (≥70%) and verapamil hydrochloride were obtained from Sigma-Aldrich (St. Louis, MO, USA). GF120918 hydrochloride salt was obtained from Glaxo-Wellcome (Research Triangle, NC, USA). Gentamicin sulfate and methyl-β-cyclodextrin were obtained from Acros Organics (Morris, NJ, USA). Triton X-100 was obtained from Integra Chemical (Kent, WA, USA). Dimethylsulfoxide (DMSO) was from MP Biomedicals (Santa Ana, CA, USA) and ethanol (200 proof) was from Pharmco-Aaper (Shelbyville, KY, USA).

Of the inhibitors used, stock solutions of filipin complex and GF120918 were prepared in DMSO whereas amiloride, chlorpromazine, colchicine and verapamil were prepared in purified water. All the final concentrations were prepared in cell culture medium. Stock solutions of gentamicin and methyl-β-cyclodextrin were directly solubilized in cell culture medium to obtain the required concentrations.

### 2.2 WST-1 and LDH Assay for Inhibitors

BeWo b30 cells were maintained in Dulbecco’s Modified Eagle’s Medium/Ham’s F-12 50/50 mixture without phenol red (Mediatech, Manassas, VA, USA) containing 10% fetal bovine serum (Atlanta Biologicals, Flowery Branch, GA, USA), antibiotic/antimycotic containing 10,000 units/mL penicillin, 10,000 μg/mL streptomycin and 25 μg/mL amphotericin B (Gibco, Carlsbad, CA, USA), MEM nonessential amino acid solution (Sigma, St. Louis, MO, USA), and 200 mM L-glutamine (Cellgro, Tewksbury, MA, USA). Cells from passage 31 (WST-1) and 26 (LDH) were seeded in a 96-well plate at a cell density of 12,500 cells/cm^2^ with 100 μL of cell culture medium per well and were allowed to grow for 48 hours. Incubation was at 37°C, 5% CO_2_ and 95% relative humidity unless otherwise specified. 48 hours post-seeding, the cell culture medium was aspirated and the endocytosis or efflux inhibitors amiloride (500 μM), chlorpromazine (10 μg/mL), colchicine (1 mM), filipin complex (3 μg/mL), gentamicin (2 mM), GF120918 (1 μM), methyl-β-cyclodextrin (5 mM), and verapamil (100 μM) prepared in phenol red-free medium were added. Cell culture medium without phenol red and 0.1% (v/v) Triton X-100 were used as positive and negative controls, respectively. Separately, cells were treated with dimethyl sulfoxide (DMSO) and ethanol at 0.01% and 0.1% (v/v) for 4 hours. At the end of the incubation period, test treatments and controls were aspirated and cells were washed once with 100 μL of phenol red free medium. After this step, the cells were tested using either the WST-1 or the LDH assay.

For the WST-1 assay (Roche Diagnostics, Mannheim, Germany), 110 μL of working solution was added to each well and further incubated for 2 hours. At the end of the incubation period, the plate was shaken for 1 minute in a Vmax^®^ microplate reader (Molecular Devices Corporation, Sunnyvale, CA, USA) and absorbance was measured at 450 nm, with subtraction of nonspecific background at 650 nm. In the LDH assay, at the end of the incubation period, plates were centrifuged at 250×*g* for 10 minutes. Carefully, 100 μL of supernatant was collected and transferred to another clear 96-well flat bottom plate and 100 μL of reaction mixture (solution C) from the LDH assay kit (Clontech Laboratories, Mountain View, CA, USA) was added to each well and left for 30 minutes without shaking at room temperature, protected from light. Absorbance was then measured at 490 nm minus the reference at 650 nm. Cytotoxicity was calculated using the following equation [6]:

$$\mathrm{Cytotoxicity}=\frac{\begin{array}{c} \mathrm{Absorbance from test treatment}-\\ \mathrm{Absorbance from cell culture medium} \end{array}}{\begin{array}{c} \mathrm{Absorbance from}\;0.1\%\;(\upsilon /\upsilon )\mathrm{Triton X}­100-\\ \mathrm{Absorbance from cell culture medium} \end{array}}\times 100\%$$

### 2.3 Statistical Analysis

Data were analyzed by one-way ANOVA with post hoc Tukey test using SigmaPlot (version 13). *P* values less than 0.05 were considered statistically significant.

## 3. RESULTS AND DISCUSSION

**Fig. 1:**
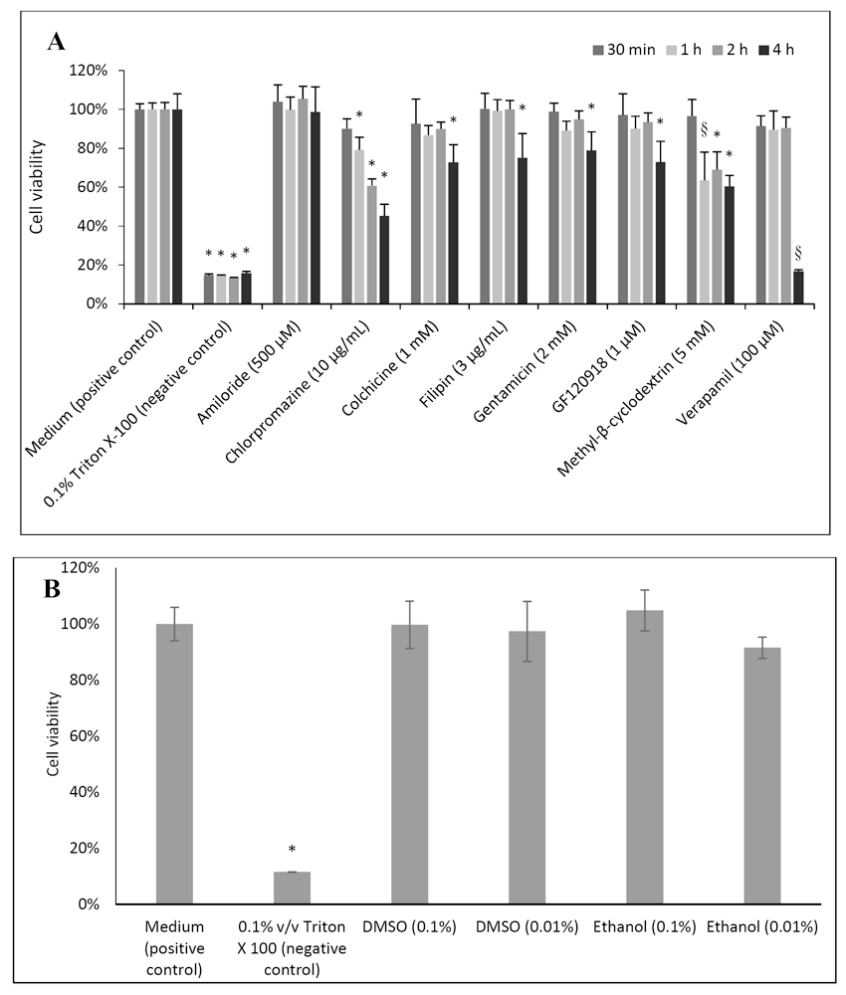
BeWo cell viability after treatment at various time points with selected endocytosis and efflux inhibitors (A) and solvents after 4 h (B), as measured by the WST-1 assay. Error bars indicate standard deviation (n=6 per group except for methyl β-cyclodextrin at 1 h, medium and verapamil at 2 h, and verapamil at 4 h, for which n=5) *indicates P<0.05 by ANOVA, and § indicates P<0.05 by ANOVA, but with a failed Brown-Forsythe equal variance test.

As expected, cells treated with 0.1% (v/v) Triton X-100 (the negative control) exhibited the lowest cell viability according to the WST-1 assay (Fig. 1). When cells were incubated with chlorpromazine, cell viability was found to be significantly reduced after 1 hour as compared to the cell culture medium (the positive control). Cell viability was also significantly decreased after 4 hours for all of the inhibitors studied except for amiloride. Cell viability was not affected after 4 hours of cell exposure to either concentration of ethanol or DMSO (both 0.01% and 0.1% v/v were studied).

**Fig. 2:**
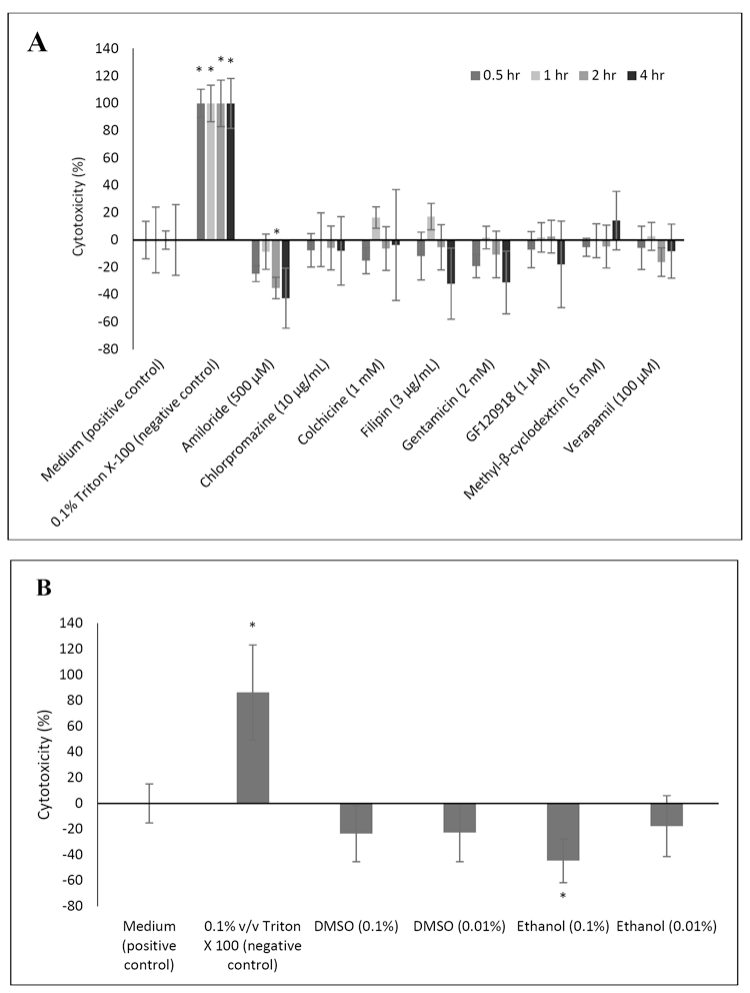
BeWo cell cytotoxicity as determined by the LDH assay following exposure of the cells to endocytosis or efflux inhibitors for various time points (A) and solvents for 4 h (B). Error bars indicate standard deviation (n=6 per group, except for medium at 1 h and 0.01 % DMSO, for which n=5) * indicates P<0.05 by ANOVA

Complementary to the WST-1 assay, cell cytotoxicity was determined using the LDH assay. In this test, the cytotoxicity of Triton-X-100 (the negative control) was set at 100% following the aforementioned equation, and accordingly the cytotoxicity of cell culture medium (the positive control) was set at zero. Fig. 2 shows that no significant cytotoxicity was observed in the BeWo cells for any of the inhibitors or solvents investigated over 4 hours. In several instances, the average absorbance values obtained were lower than the average absorbance readings of the cell culture medium, thus resulting in slightly negative values as per the equation. Therefore, the significant decrease of cytotoxicity in the case of amiloride at 2 hours and of 0.01% ethanol at 4 hours do not raise concerns for cellular membrane integrity. Comparing Figs. 1 and 2, exposure of BeWo cells to these compounds appears to elicit decreases in cell viability at concentrations and times of exposure that do not yet elicit corresponding decreases in cytotoxicity. The WST-1 and LDH assays measure different aspects of cellular function. The WST-1 assay (like the MTT assay) quantifies mitochondrial activity, but it is not indicative of apoptosis [13]. It is possible that BeWo cell mitochondrial activity is compromised prior to cellular membrane disruption and cell death associated with the release of LDH.

The observed lack of cytotoxicity for DMSO and ethanol up to 0.1% (v/v) by both the WST-1 and LDH assays at 4 hours are in good agreement with the report of Jamalzadeh et al. [14], who observed no significant decreases in cell viability upon exposure of RAW 264.7, MCF-7, and human umbilical vein endothelial cells to 0.1% DMSO and 0.1% ethanol. However, cell viability was reported to decrease as the solvent concentrations increased.

## 4. CONCLUSION

Depending upon the desired time points for mechanistic cellular uptake and transport studies, suitable endocytosis and efflux inhibitors should be selected carefully so that they do not impart any toxicity to the cells nor reduce their metabolic activity. Of the inhibitors investigated herein, the use of chlorpromazine or methyl-β-cyclodextrin beyond 30 minutes (or most of these inhibitors for 4 hours) may adversely affect studies of endocytosis or efflux mechanisms in BeWo cells. It should be mentioned that the concentration studied for each inhibitor was based on literature reports, and it is possible that lower concentrations of these inhibitors might not reduce cell viability to the same extent while still maintaining the desired inhibitory action. Based on these results, it is important to ensure that compounds utilized to elucidate endocytosis or efflux mechanisms do not adversely affect cellular functions in a manner that could compromise experimental results and conclusions.

## ABBREVIATIONS

WST-1: water-soluble tetrazolium-1
LDH: lactate dehydrogenase
